# Cancer Wars: Significance of Protein Unfolding in Cancer and Its Inhibition with Natural Amphiphilic Substances

**DOI:** 10.3389/fonc.2014.00183

**Published:** 2014-07-11

**Authors:** Boguslaw Lipinski

**Affiliations:** ^1^Joslin Diabetes Center, Harvard Medical School, Boston, MA, USA

**Keywords:** cancer, fibrinogen, iron, polyphenols, protein disulfide bonds, selenium

It is essential to bear in mind that the native conformation of human proteins is stabilized by *intra*-molecular disulfide (S–S) bonds between a single or multiple polypeptide chains. The formation of S–S bonds is catalyzed by protein disulfide isomerase (PDI) (1), the activation of which is associated with a number of human diseases, such as myocardial infarction, stroke, and cancer. Unless proper chaperone proteins are available the accidental cleavage of S–S linkages will result in the unfolding and scrambled refolding of the polypeptide chains thus producing non-native species present in many degenerative diseases (2–5). This process is the most common protein post-translational modification that, for example, in a protein with 9 disulfide bridges can theoretically form 34,459,425 different disulfide-bonded isomers, only one with a correct protein function. However, the most damaging consequence of such a modification is the formation of protein aggregates known as “inclusion bodies” that are resistant to the enzymatic degradation (6). This unusual phenomenon is the result of the formation of *inter*-molecular hydrophobic bonds, which in contrast to peptide links are not susceptible to the catalytic hydrolysis. It is known that the strongest and practically irreversible protein interactions involve hydrophobic bonds, which in native proteins are buried inside their tertiary structure (7).

One of the blood proteins rich in disulfides is fibrinogen (Fbg), the physiologic function of which is to provide hemostatic fibrin plug formed by the action of enzyme thrombin. This insoluble polymer, when formed at the site of vessel wall injury, is eventually removed by the action of fibrinolytic enzyme system, to give space for the growth of a connective tissue and to ensure proper wound healing. To speed up the process of fibrin elimination from the coronary or cerebral circulations, several thrombolytic therapies have been devised with the use of a variety of fibrinolytic agents. It is, however, well recognized in clinical practice that such therapies are effective only when installed 3–5 h after the onset of thrombosis (8). This enigma is now resolved by the discovery of the alternative pathway of blood coagulation induced with iron (9). Thus, in contrast to thrombin-generated fibrin the iron-induced *parafibrin* is totally resistant to the fibrinolytic degradation. This is due to the fact that parafibrin has different a tertiary structure than fibrin formed with thrombin. We have showed that such a dramatic modification of fibrin structure is due to the unfolding and scrambled refolding of Fbg disulfide-linked subunits leading to the exposure of hydrophobic epitopes in their polypeptide chains (9). The cleavage of disulfide bonds is induced by biologically highly reactive hydroxyl radicals (HO^⋅^) formed in the reaction between trivalent iron with hydroxyl groups of water according to the following formula:

$$\text{F}{\text{e}}^{3+}+\text{H}{\text{O}}^{-}\to \text{F}{\text{e}}^{2+}+\text{H}{\text{O}}^{•}.$$

As the consequence of the hydroxyl radical interaction with Fbg a huge protease resistant polymer is formed that remains in the circulation for a long time, resulting in a state of chronic inflammation due to the attraction of cytotoxic albeit ineffective T cells. The accumulating evidence indicates that there is a correlation between increased blood concentration of unbound iron and the incidence of cancer in humans (10–13), and that its reduction may prevent cancer morbidity and mortality (14). It should be noted that it is only the trivalent iron (Fe^3+^), and not divalent (Fe^2+^), which participate in the generation of hydroxyl radicals and subsequent formation of insoluble parafibrin from soluble plasma Fbg. However, when hemoglobin is released from the hemolyzed erythrocytes, the divalent ferrous ions are enzymatically converted to ferric ions. Thus, any pathologic condition in which erythrocyte membranes are damaged, e.g., in infections and/or after exposure to environmental toxins, may contribute to the excessive body storage of trivalent iron. It should be borne in mind that this form of iron accumulates with age due to the fact that there is no mechanism for its physiologic elimination, and may therefore, explain association of cancer with aging.

The unsuccessful attempts at removing parafibrin by the human body defense systems were recently suggested to contribute to Alzheimer’s disease (15) as well as to the cardiovascular disease (16). These diseases and other degenerative disorders have been known to respond well to dietary modifications, particularly to those associated with the so-called Mediterranean diet (17), which is rich in natural amphiphilic substances such as polyphenols and flavones. Relevant to the concept presented in this article is the fact that protein unfolding can be inhibited by natural products present in tea, fruits, berries, and certain grains, the consumption of which is known to lower the incidence of degenerative diseases (18). In addition, there is another important component of human diet, selenium, which is known to prevent various forms of cancer (19–23). Hence, sodium selenite, but not selenate, reacts with free sulfhydryl groups of proteins, thus preventing reductive cleavage of disulfide bonds followed by protein unfolding and abnormal refolding (24, 25).

It is proposed in this paper that the barrier formed around tumor cells composed of proteolytically resistant parafibrin can be removed by a non-enzymatic mechanism based on the interaction of hydrophobic and hydrophilic groups (Figure 1). Numerous natural substances, particularly those of amphiphilic nature such as polyphenols, when ingested with diet in sufficient quantities can prevent and/or reverse cancer formation and metastases (26–33). These findings may explain beneficial effects of the Mediterranean diet known to be associated with lower incidence of cancer and other degenerative diseases (34).

**Figure 1:**
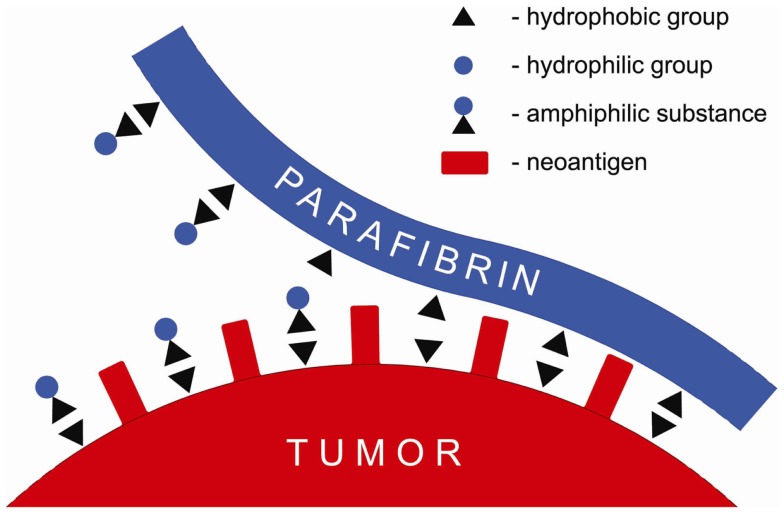
**Schematic presentation of a putative mechanism of the anticancer effect of amphiphilic substances that displace parafibrin from surface of the cancer cell membrane**.

According to the mechanism shown in Figure 1 amphiphilic substances taken up with food interfere with and/or displace the hydrophobic coat on cancer cells membranes thus exposing neoantigens and making tumors vulnerable to the natural killer cells attack and lysis.

In conclusion, the concept delineated in this article supports the original notion expressed by Basmadijan et al. (35) according to which natural products have a potential to be developed into novel anticancer medicines.

## Conflict of Interest Statement

The author declares that the research was conducted in the absence of any commercial or financial relationships that could be construed as a potential conflict of interest.

## References

[1] Gilbert HF Protein disulfide isomerase and assisted protein folding. J Biol Chem (1997) 272:29399–402 10.1074/jbc.272.14.8845 9367991

[2] Anfinsen CB Principles that govern the folding of protein chains. Science (1973) 181:223–30 10.1126/science.181.4096.223 4124164

[3] Cook KM Hogg PJ Post-translational control of protein function by disulfide bond cleavage. Antioxid Redox Signal (2013) 18:1987–2015 10.1089/ars.2012.4807 23198756

[4] Gregersen N Bross P Vang S Christensen JH Protein misfolding and human disease. Annu Rev Genomics Hum Genet (2006) 7:103–24 10.1146/annurev.genom.7.080505.115737 16722804

[5] Wang S Kaufman RJ The impact of the unfolded protein response on human disease. J Cell Biol (2012) 197:857–67 10.1083/jcb.201110131 22733998 PMC3384412

[6] Fahnert B Lilie H Neubauer P Inclusion bodies: formation and utilisation. Adv Biochem Eng Biotechnol (2004) 89:93–142 10.1007/b93995 15217157

[7] Lins L Brasseur R The hydrophobic effect in protein folding. FASEB J (1995) 9:535–40 7737462 10.1096/fasebj.9.7.7737462

[8] Lipinski B Modification of fibrin structure as a possible cause of thrombolytic resistance. J Thromb Thrombolysis (2010) 29:296–8 10.1007/s11239-009-0367-6 19551349

[9] Lipinski B Pretorius E Novel pathway of iron-induced blood coagulation: implications for diabetes mellitus and its complications. Pol Arch Med Wewn (2012) 122:115–22 22460041

[10] Nelson RL Dietary iron and colorectal cancer. Free Radic Biol Med (1992) 12:161 10.1016/0891-5849(92)90010-E 1559619

[11] Norat T Lukanova A Ferrari P Riboli E Meat consumption and colorectal cancer risk; dose-response meta-analysis of epidemiological studies. Int J Cancer (2002) 98:241–6 10.1002/ijc.10126 11857415

[12] Steegmann-Olmedillas JL The role of iron in tumor cell proliferation. Clin Transl Oncol (2011) 13:71–6 10.1007/s12094-011-0621-1 21324793

[13] Toyokuni S Role of iron in carcinogenesis: cancer as a ferrotoxic disease. Cancer Sci (2009) 100:9–16 10.1111/j.1349-7006.2008.01001.x 19018762 PMC11158384

[14] Kalinowski DS Richardson DR The evolution of iron chelators for the treatment of iron overload disease and cancer. Pharmacol Rev (2005) 57:547–83 10.1124/pr.57.4.2 16382108

[15] Lipinski B Pretorius E The role of iron-induced fibrin in the pathogenesis of Alzheimer’s disease and the protective role of magnesium. Front Hum Neurosci (2013) 7:735 10.3389/fnhum.2013.00735 24194714 PMC3810650

[16] Lipinski B Pretorius E Iron-induced fibrin in cardiovascular disease. Curr Neurovasc Res (2013) 10:269–74 10.2174/15672026113109990016 23721262 PMC3763776

[17] Schwingshacki L Hoffmann G Adherence to Mediterranean diet and risk of cancer: a systematic review and meta-analysis of observational studies. Int J Cancer (2014). 10.1002/ijc.28824 24599882

[18] Lipinski B Hydroxyl radical and its scavengers in health and disease. Oxid Med Cell Longev (2011) 2011:809696 10.1155/2011/8099696 21904647 PMC3166784

[19] Lipinski B Prostate cancer vaccines, fibrin and selenium: a conceptual review. Open Prost Cancer J (2010) 3:69–73 10.2174/1876822901003010069

[20] Freitas M Alves V Sarmento-Ribeiro AB Mota-Pinto MA Combined effect of sodium selenite and docetaxel on PC3 metastatic prostate cancer cell line. Biochem Biophys Res Commun (2011) 408:713–9 10.1016/j.bbrc.2011.04.109 21549092

[21] Micke O Schomburg L Buentzel J Kisters K Muecke R Selenium in oncology: from chemistry to clinics. Molecules (2009) 14:3975–88 10.3390/molecules14103975 19924043 PMC6255034

[22] Rayman MP Selenium in cancer prevention: a review of the evidence and mechanism of action. Proc Nutr Soc (2005) 64:527–42 10.1079/PNS2005467 16313696

[23] Kralova V Brigulova K Cervinka M Rudolf E Antiproliferative and cytotoxic effects of sodium selenite in human colon cancer. Toxicol In vitro (2009) 23:1497–503 10.1016/j.tiv.2009.07.012 19602434

[24] Frenkel GG Falvey D MacVicar C Products of the reaction of selenite with intracellular sulfhydryl groups. Biol Trace Elem Res (1991) 30:9–18 10.1007/BF02990338 1718373

[25] Spallholtz JE On the nature of selenium toxicity and carcinostatic activity. Free Radic Biol Med (1994) 17:45–64 10.1016/0891-5849(94)90007-8 7959166

[26] Adhami VM Mukhtar H Polyphenols from green tea and pomegranate for prevention of prostate cancer. Free Radic Res (2006) 40:1095–104 10.1080/10715760600796498 17015254

[27] Fresco P Borges F Diniz C Marques MP New insight on the anticancer properties of dietary polyphenols. Med Res Rev (2006) 26:747–66 10.1002/med.20060 16710860

[28] Heger M van Golen RF Broekgaarden M Michel MC The molecular basis for the pharmacokinetics and pharmacodynamics of curcumin and its metabolites in relation to cancer. Pharmacol Rev (2014) 66:222–307 10.1124/pr.110.004044 24368738

[29] Lambert JD Elias RJ The antioxidant and pro-oxidant activities of green tea polyphenols: a role in cancer prevention. Arch Biochem Biophys (2010) 501:65–72 10.1016/j.abb.2010.06.013 20558130 PMC2946098

[30] Radin NS Meta-analysis of anticancer drug structures – significance of their polar allylic moieties. Anticancer Agents Med Chem (2007) 7:209–22 10.2174/187152007780058696 17348828

[31] Schramm L Going green: the role of the green tea component EGCG in chemoprevention. J Carcinog Mutagen (2013) 4(142):1000142 10.4172/2157-2518.1000142 24077764 PMC3783360

[32] Shukla Y Singh R Resveratrol and cellular mechanisms of cancer prevention. Ann N Y Acad Sci (2011) 1251:1–8 10.1111/j.1749-6632.2010.05870.x 21261635

[33] Takemura H Sakakibara H Yamazaki S Shimoi K Breast cancer and flavonoids – a role in prevention. Curr Pharm Des (2013) 19:6125–32 10.2174/1381612811319340006 23448447

[34] Bosetti C Turati F Dal Pont A Ferraroni M Polesel J Negri E The role of Mediterranean diet on the risk of pancreatic cancer. Br J Cancer (2013) 109:1360–6 10.1038/bjc.2013.345 23928660 PMC3778270

[35] Basmadijan C Zhao Q Bentouhami E Djehal A Negibil CG Johnson RA Cancer wars: natural products strike back. Front Chem (2014) 2:20 10.3389/fchem.2014.00020 24822174 PMC4013484

